# A Mechanism for Actin Filament Severing by Malaria Parasite Actin Depolymerizing Factor 1 via a Low Affinity Binding Interface* [Fn FN2]

**DOI:** 10.1074/jbc.M113.523365

**Published:** 2013-12-26

**Authors:** Wilson Wong, Andrew I. Webb, Maya A. Olshina, Giuseppe Infusini, Yan Hong Tan, Eric Hanssen, Bruno Catimel, Cristian Suarez, Melanie Condron, Fiona Angrisano, Thomas NebI, David R. Kovar, Jake Baum

**Affiliations:** From the Divisions of ‡ Infection and Immunity and; ¶ Systems Biology and Personalised Medicine, The Walter and Eliza Hall Institute of Medical Research, Parkville, Victoria 3052, Australia,; the § Department of Medical Biology, University of Melbourne, Parkville, Victoria 3052, Australia,; the ‖ Electron Microscopy Unit, Bio21 Molecular Science and Biotechnology Institute, University of Melbourne, Parkville, Victoria 3010, Australia,; the Departments of ‡‡ Molecular Genetics and Cell Biology and; §§ Biochemistry and Molecular Biology, University of Chicago, Chicago, Illinois 60637, and; the ** Ludwig Institute for Cancer Research, Austin Hospital, Olivia Newton-John Cancer and Wellness Centre, Heidelberg, Victoria 3084, Australia

**Keywords:** Actin, Cofilin, Cytoskeleton, Electron Microscopy (EM), Malaria, Mass Spectrometry (MS), Plasmodium, Protein Cross-linking

## Abstract

Actin depolymerizing factor (ADF)/cofilins are essential regulators of actin turnover in eukaryotic cells. These multifunctional proteins facilitate both stabilization and severing of filamentous (F)-actin in a concentration-dependent manner. At high concentrations ADF/cofilins bind stably to F-actin longitudinally between two adjacent actin protomers forming what is called a decorative interaction. Low densities of ADF/cofilins, in contrast, result in the optimal severing of the filament. To date, how these two contrasting modalities are achieved by the same protein remains uncertain. Here, we define the proximate amino acids between the actin filament and the malaria parasite ADF/cofilin, PfADF1 from *Plasmodium falciparum.* PfADF1 is unique among ADF/cofilins in being able to sever F-actin but do so without stable filament binding. Using chemical cross-linking and mass spectrometry (XL-MS) combined with structure reconstruction we describe a previously overlooked binding interface on the actin filament targeted by PfADF1. This site is distinct from the known binding site that defines decoration. Furthermore, total internal reflection fluorescence (TIRF) microscopy imaging of single actin filaments confirms that this novel low affinity site is required for F-actin severing. Exploring beyond malaria parasites, selective blocking of the decoration site with human cofilin (HsCOF1) using cytochalasin D increases its severing rate. HsCOF1 may therefore also use a decoration-independent site for filament severing. Thus our data suggest that a second, low affinity actin-binding site may be universally used by ADF/cofilins for actin filament severing.

## Teaser

**Background:** *Plasmodium falciparum* actin depolymerizing factor 1 (PfADF1) severs actin polymers without stable filament-binding, challenging current models for severing.

**Results:** Cross-linking mass spectrometry of PfADF1 with filamentous actin reveals a novel binding interface required for severing.

**Conclusion:** Filament severing by PfADF1 is via a previously unidentified binding interface.

**Significance:** We propose an alternative mechanism for actin filament severing potentially used across eukaryotic cells.

## Introduction

Rapid reorganization of the actin cytoskeleton lies at the heart of numerous cellular processes within the eukaryotic cell (1). A primary class of regulator that controls this turnover is the actin depolymerizing factor (ADF)^5^/cofilin family of proteins (2, 3). One of their key functions is actin filament (F-actin) severing, which produces additional free ends for filament growth or leads to complete polymer disassembly (4, 5), both of which underpin actin-dependent processes such as cell movement (2, 3).

ADF/cofilin proteins are multifunctional regulators, able to bind monomeric (G)-actin or filamentous (F)-actin, as well as modulate the state of actin in a concentration-dependent manner (6), although the mechanism for this diverse (and contrasting) functionality is not known. At nanomolar concentrations ADF/cofilins preferentially mediate filament severing via low density binding to F-actin (6, 7). At micromolar concentrations the filament becomes saturated with ADF/cofilins leading to a stabilized F-actin or cofilactin polymer (6), whereas at very high concentrations (several micromolar) filament nucleation can occur (6). Stable ADF/cofilin binding to the F-actin polymer leads to a twist and reduced pitch along its length (8) and is thought to partly explain the ability of ADF/cofilins to both sever actin filaments and mediate their stabilization. Recent cryoelectron microscopy (cryo-EM) structural analysis of human cofilin 2 (HsCOF2)-decorated filaments detailed the cofilin-F-actin interaction as stabilized between two adjacent actin protomers along the longitudinal axis of the filament (9). Interaction with the upper protomer (*n*+2) is focused between subdomains (SD) 1 and SD3 of the actin monomer, whereas interaction with SD2 of the adjacent actin monomer (protomer n) in the filament is mediated by a domain comprising the protruded β hairpin and α4 helix of HsCOF2, referred to as the ADF/cofilin F-site (9). This combined interaction is thought to lead to a conformational shift in SD2 of the protomer *n*, resulting in a twist along the actin polymer. Combined with biophysical data demonstrating that decorated filaments have enhanced elasticity, this has led to a rational theoretical model in which mechanical asymmetry at boundaries of bare and decorated filament segments leads to an accumulation of stress thereby promoting severing (10). Recent two-color total internal reflection fluorescence (TIRF) microscopy with *Saccharomyces cerevisiae* cofilin1 (ScCOF) has provided experimental support for severing at these boundary sites (11). Of note, the interaction of HsCOF2 with SD1 and SD3 of the *n*+2 actin protomer in the cryo-EM filament is structurally conserved with that identified from the co-crystal structure of monomeric actin bound to the non-severing (and non-filament binding) C-terminal ADF/cofilin homology domain of mouse twinfilin (12). Monomer binding and filament decoration therefore appear to be structurally linked. As such, current understanding favors a model in which multifunctionality of ADF/cofilins is explained as concentration-dependent manifestations of filament decoration (13).

In apicomplexan parasites, single-celled protozoan pathogens that include the etiological agents of malaria and toxoplasmosis, ADF/cofilin proteins display unusual properties in their regulation and interaction with the actin filament (14–18). They are able to sever filaments yet possess low binding affinity for F-actin (15, 18). The reduction in F-actin binding affinity is explained by the marked reduction of the filament binding loop and α4 helix (F-site) in apicomplexan ADFs (17–19). Thus, comparative investigation of the molecular mechanism of F-actin binding by divergent ADF/cofilins, including those from apicomplexan parasites, may reveal a common mechanism that governs severing across all ADF/cofilins.

Here, we describe a novel mechanism for F-actin severing by PfADF1 via low affinity binding to a decoration-independent binding interface. Combining chemical cross-linking and mass spectrometry (XL-MS) with protein complex structure reconstruction, we have built structural models of malaria parasite, *Plasmodium falciparum*, ADF1 in complex with vertebrate G- and F-actin. Contrary to expectations, PfADF1 interacts with the actin polymer via actin residues that are not involved in canonical F-actin decoration by ADF/cofilins. Furthermore, single molecule imaging with PfADF1 mutants reveals the key role of residues involved in this novel interaction for F-actin severing. Exploring the conservation of this interaction, we also demonstrate that selective chemical inhibition of the classical decoration site leads to an enhancement in severing by human cofilin 1 (HsCOF1). This suggests a mechanism whereby HsCOF1 has been forced to interact with a site independent of the canonical decoration site. We, therefore, propose that the novel mechanism used by malaria parasite PfADF1 is potentially employed by diverse ADF/cofilins to mediate rapid F-actin turnover.

## EXPERIMENTAL PROCEDURES

### 

#### 

##### Protein Expression, Purification, and Biochemical Assays

Recombinant ADF/cofilins were expressed as GST fusion proteins from BL21(DE3) strain of *Escherichia coli* using the pGEX4T vector (GE Healthcare). GST was cleaved using thrombin protease (GE Healthcare) followed by size-exclusion chromatography (Superdex 200, GL 10/300 column; GE Healthcare) to purify untagged proteins. Rabbit skeletal muscle actin was used in all experiments either from a commercial source (Cytoskeleton Inc.) or purified from muscle tissue by conventional methods.

##### Electron Microscopy

Recombinant ADF/cofilins were added to 2 μm pre-formed rabbit skeletal muscle actin filaments for 30 min. Solutions were adsorbed on formvar-carbon films supported on 200-mesh copper grids. Grids were glow discharged before being negatively stained with aqueous uranyl acetate. Samples then were observed with an FEI Tecnai F30 microscope. Filament length and pitch were measured (blind for each sample) using the Segmented Lines tool from ImageJ.

##### Biochemical Assays

All ADF/cofilins (GST removed) used for biochemical assays were purified in the elution buffer (20 mm MES, pH 7, 10 mm NaCl) used for size exclusion chromatography. In all biochemical assays, actin (2 μm) in G-buffer (20 mm MES, pH 7, 0.1 mm ADP, and 0.1 mm CaCl_2_) was induced to polymerize by the addition of ×10 polymerization buffer (1 m KCl and 20 mm MgCl_2_). Actin sedimentation analysis was used to measure F-actin severing or G-actin sequestration by ADF/cofilin proteins with either F- or G-actin as input, respectively. For severing, F-actin (2 μm) was incubated with various concentrations of ADF/cofilins for 1 h. Sequestration assays were performed by incubating G-actin with various concentrations of ADF/cofilins for 1 h. Samples were ultracentrifuged at 100,000 × *g* (TLA 100 rotor, Beckman Coulter Optima TL Ultracentrifuge) for 1 h, washed, and re-centrifuged before pellets were resuspended in an equal volume of reducing SDS-PAGE sample buffer. Equal amounts of supernatant and pellet fractions were analyzed by SDS-PAGE and quantification of protein was performed by densitometry analysis using a GS-800 calibrated densitometer (Bio-Rad).

##### TIRF Microscopy

TIRF images of a mixture of 1.0 μm Mg-ATP/actin supplemented with 0.5 μm Oregon Green-labeled Mg-ATP/actin excited by evanescent wave fluorescence were acquired every 10 s on an IX-71 microscope (Olympus) fit with through the objective TIRF illumination and an iXon EMCCD camera (Andor Technology), as described (20). Filaments were assembled until they reached >10 μm in length. For experiments with PfADF1/PfADF1.K100AD120A, 1.6 chamber volumes (16 μl) of protein diluted in TIRF buffer (10 mm imidazole, pH 7.0, 50 mm KCl, 1 mm MgCl_2_, 1 mm EGTA, 100 mm DTT, 0.2 mm ATP, 15 mm glucose, 20 μg/ml of catalase, 100 μg/ml of glucose oxidase, 0.5% methylcellulose (4000 cp)) were introduced into the chamber by capillary action during continuous imaging. For experiments with cytochalasin D (CD) (Sigma C2618), 16 μl of DMSO or CD diluted in TIRF buffer were introduced into the chamber. After ∼2 min, 16 μl of PfADF1 or HsCOF1 diluted in TIRF buffer were introduced secondarily. Final protein concentrations are as indicated in figures or text.

##### Surface Plasma Resonance

Interaction studies between PfADF1-WT and PfADF1.K72A with G-actin were conducted using a BIAcore 2000 biosensor (BIAcore 2000, Uppsala, Sweden). Actin was immobilized onto a linear polycarboxylate hydrogel sensor chip (Xantec Bioanalytics, Germany) using NHS/EDC chemistry. Binding was performed in G-buffer (20 mm MES, pH 7, 0.1 mm CaCl_2_, and 0.1 mm ADP). Various concentrations of PfADF1-WT (69.30, 34.65, 17.32, 8.66, and 4.33 μm) and PfADF1.K72A (68.70, 34.35, 17.18, 8.60, and 42.95 μm) were injected over immobilized actin (4.2 ng/mm^2^ immobilized). A derivatized blank channel was used as control. Kinetic constants were derived from the resulting sensorgrams with BIAevaluation 4.1 software (Biacore Life Sciences, GE Healthcare) using Global analysis using a 1:1 model that includes terms for mass transfer of analyte to the surface. Affinity constants were not calculated at steady state as the low association rates of the interactions between actin and PfADF1 proteins would require large analyte consumption. This 1:1 interaction model is equivalent to the Langmuir isotherm for adsorption to a surface. Data were fitted using a global analysis module that constrains selected parameters (association rate (*k_a_*) and dissociation rate (*k_d_*)) to a single solution for all sets of binding curves, improving the robustness of the fitting procedure (21). This set of rate constants was used for calculation of the equilibrium dissociation constant (*K_D_* = *k_d_*/*k_a_*).

##### XL-MS and Pseudo-structure Reconstruction

Recombinant ADF/cofilins (50 μm) were incubated with G-actin (24 μm) in the presence of sulfo-SDA or EDC in G-buffer for 0.5 or 2 h, respectively. For F-actin-ADF/cofilin cross-linking, G-actin was first induced to polymerize by the additional 1/10th volume of ×10 F-buffer (500 mm KCl, 20 mm MgCl_2_) for 1 h at room temperature. EDC and ADF/cofilins were then added to F-actin and incubated at room temperature for 2 h. Cross-linked F-actin·ADF/cofilin complexes were pelleted at 100,000 × *g* in a TLA 100 rotor for 1 h before sample analysis. Sulfo-SDA-treated samples were subsequently photoactivated by UV illumination at 360 nm for 20 min. Cross-linked actin·ADF/cofilin complexes were separated by SDS-PAGE and complex bands were excised, followed by manual in-gel reduction, alkylation, and tryptic digestion. Extracted peptides were injected and separated by nano-flow reversed-phase liquid chromatography on a nano-LC system (1200 series, Agilent) using a nanoAcquity C18 150 × 0.15-mm inner diameter column (Waters) with a linear 60-min gradient set at a flow rate of 1.2 μl/min at 45 °C from 100% solvent A (0.1% formic acid in Milli-Q water) to 100% solvent B (0.1% formic acid, 60% acetonitrile (Mallinckrodt Baker, NJ), 40% Milli-Q water). For SDA cross-linked samples, the nano-HPLC was coupled on-line to an LTQ-Orbitrap XL mass spectrometer equipped with a nanoelectrospray ion source (Thermo Fisher Scientific) for automated MS/MS. The Orbitrap was run in a data-dependent acquisition mode with the Orbitrap resolution set at 60,000 and the top five multiply charged species selected for fragmentation in the linear ion trap by collision-induced dissociation (single charged species were ignored). The ion threshold was set to 15,000 counts for MS/MS. The activation time was set to 30 ms. For EDC cross-linked samples, digested samples were injected and separated by nano-flow reversed-phase liquid chromatography on a nano LC system (Waters nanoAcquity) using a nanoAcquity C18 150 × 0.075-mm inner diameter column (Waters) with a linear 60-min gradient set at a flow rate of 0.4 μl/min from 95% solvent A (0.1% formic acid in Milli-Q water) to 100% solvent B (0.1% formic acid, 80% acetonitrile (Mallinckrodt Baker, NJ), 20% Milli-Q water). The nano-UPLC was coupled on-line to a Q-Exactive mass spectrometer equipped with a nano-electrospray ion source (Thermo Fisher Scientific) set to acquire full scan (70,000 resolution) and the top 10 multiply charged species selected for fragmentation using the high energy collision disassociation (single charged species were ignored). Fragment ions where analyzed with resolution set at 17,500 with the ion threshold set to 1e5 intensity. The activation time was set to 30 ms and the normalized collision energy set to 24. Raw files consisting of full-scan MS, low-resolution MS/MS (OrbiTrap XL), and high-resolution MS/MS spectra (Q-Exactive) were converted to the MGF data format with Proteome Discoverer 1.4 and searched using Xcomb generated databases with the Mascot or pLink algorithms, respectively (22). Databases for every possible cross-linked pair were generated by xComb version 1.3 (23). The amino acid sequences for the expressed recombinant ADF proteins were derived from its genetic sequence and the Uniprot entry for rabbit actin were uploaded in Uniprot FASTA format. Trypsin (two missed cleavages) was chosen for enzyme digestion. Both intra- and inter-protein cross-links were created. The minimum peptide length for each peptide of the pair was four amino acids with at least one trypsin missed cleavage selected with amine cross-linking. The cross-link database was uploaded in Mascot version 2.3. Mascot parameters for each search included no enzyme cleavages and a fixed modification in the form of carboxymethyl at Cys residues. Spectra were searched with a mass tolerance of 20 ppm in MS mode and 0.5 Da in MS/MS mode for the Orbitrap XL. The MS/MS fragmentation of cross-linked peptides identified by the Mascot search was manually analyzed to assign ion peaks using mMass (24) and annotated with assistance from the recently published Expert System GUI as a guide (25). For high resolution MS/MS Q-Exactive data, we searched for cross-linked peptides using the pLink algorithm (22). The database consisted of the Uniprot annotated entries for all recombinant proteins used and pLink configuration set default mass accuracy settings. Cross-linker settings were also edited to include two EDC entries (−18.0152 Da) for Lys linked to Glu and Asp residues. XL-MS determined cross-linked peptide interfaces and the crystal structures of rabbit actin (PDB code 1J6Z), PfADF1 (PDB code 3Q2B), and HsCOF1 (PDB code 1Q8X) were used to reconstruct the monomeric actin-ADF/cofilin structural models using the built in rotation and translation functions within PyMol (Delano Scientific LLC). The F-actin-PfADF1 and F-actin-HsCOF1 structural models (PDB codes 3G37 (ref) 3Q2B and 1Q8X (ref), respectively) were built using the same method.

## RESULTS

### 

#### 

##### Twist-independent Severing by PfADF1

**FIGURE 1.**
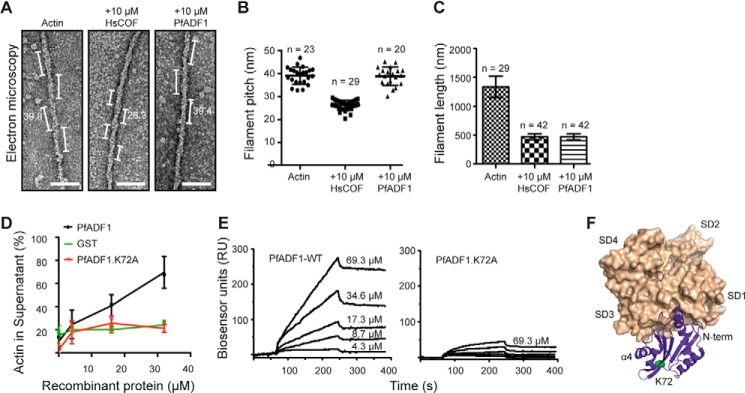
**PfADF1 interaction with monomeric and filamentous actin.** *A*, negative stain and TEM of F-actin with and without ADF/cofilin proteins. Measurements shown are in nm, with examples of pitch highlighted. *Scale bars*, 50 nm. *B*, measurement of filament pitch by TEM (mean ± S.E.). *C*, measurement of filament length by TEM (mean ± S.E.). *D*, sedimentation analysis of PfADF1 and PfADF1.K72A in preventing the formation of long filaments. G-actin (2 μm) was incubated with increasing concentrations of ADF proteins for 1 h before ultracentrifugation to separate short actin species (*Supernatant*) and polymerized F-actin (pellet). Equal amounts of supernatant and pellet fractions were assessed by SDS-PAGE and the percentage of actin in each fraction was analyzed by densitometry (*n* = 3, mean ± S.E.). *E*, measurement of binding affinities between G-actin and PfADF1 or PfADF1.K72A derivatives using surface plasma resonance. The experimental data were analyzed using a 1:1 interaction model using the rate equation described in BIAevaluation software 4.1. Data were fitted using a global analysis module that constrains selected parameters (*k_a_* and *k_d_*) to a single solution for the all set of binding curves, improving the robustness of the fitting procedure. This set of rate constants was used for calculation of the equilibrium dissociation constant (*K_D_* = *k_d_*/*k_a_*). *F*, model of published crystal structure of the Twinfilin·ADFH·actin complex (PDB code 3DAW). Serine 252 of Twinfilin-ADFH is shown as a *green sphere*, which is the equivalent residue to Lys-72 of PfADF1.

The malaria parasite *P. falciparum* ADF/cofilin (PfADF1) has low binding affinity for actin filaments (15, 18). Given PfADF1 lacks the critical motif (the filament binding loop and α4 helix comprising the F-site) thought to be required for canonical decoration, we hypothesized PfADF1 may not be able to reduce the pitch of actin filaments. To test this hypothesis, we directly visualized polymerized rabbit skeletal muscle actin by transmission electron microscopy (TEM) in the presence or absence of recombinant PfADF1 or the canonical ADF/cofilin HsCOF1, which retains an intact F-site (9). In the absence of ADF/cofilin binding, filaments demonstrated a typical ∼39.8 nm pitch (*n* = 23, Fig. 1, *A* and *B*) (8). Following incubation with 10 μm HsCOF1, filament pitch was reduced to a 26.3 nm mean, confirming known dimensions of the twisted HsCOF1-decorated filament (Fig. 1, *A* and *B*) (8). Similar concentrations of PfADF1, known to be active for severing (18), demonstrated no significant reduction in pitch (mean 39.4, *n* = 20, Fig. 1, *A* and *B*). Apicomplexan ADF/cofilin proteins thus appear unable to alter the pitch of F-actin. Despite the inability of PfADF1 to alter the pitch of the actin polymer, measurement of filament lengths by TEM showed that both HsCOF1 and PfADF1 efficiently reduced F-actin length, with a 3-fold reduction in polymer length for both proteins compared with untreated filaments (Fig. 1*C*). The ability of PfADF1 to sever was confirmed by TIRF microscopy (supplemental Movies S1 and S2) and the reduction in lengths seen are entirely consistent with previous reports of comparable severing rates, as measured by TIRF, between PfADF1 and HsCOF1 (18) along with a related ADF/cofilin from the apicomplexan parasite *Toxoplasma gondii* (15). This therefore demonstrates that actin filament severing does not require stable decoration of F-actin by PfADF1 and suggests that an alternative mechanism may exist that underpins PfADF1-mediated severing.

##### A Non-severing PfADF1 Mutant Cannot Bind G-actin

In our previous investigation of the molecular basis for filament severing by PfADF1, we showed that a structurally conserved residue at the base of the reduced F-loop, lysine 72 (Lys-72), plays a key role in filament severing (18). However, the role played by Lys-72 was not clear. To explore its activity further, we compared the effect of wild type PfADF1 and the K72A mutant on the inherent ability of G-actin to form pelletable filaments. Sedimentation assays with wild type PfADF1 demonstrated its ability to efficiently prevent the formation of long filaments in a concentration-dependent manner (Fig. 1*D*). In contrast, PfADF.K72A was unable to prevent long filaments from forming at the same concentrations tested, possibly indicating a reduced binding affinity for actin monomers or short polymers (Fig. 1*D*). Direct measurement of the actin monomer binding affinity for each ADF/cofilin protein using surface plasma resonance showed that PfADF1.K72A had 10-fold reduced affinity compared with wild type (*K_d_* for PfADF1 and PfADF1.K72A = 3.5 and 33 μm, respectively; Fig. 1*E*). In addition, the association rate constant for wild type PfADF1 was 1.5-fold higher than PfADF1.K72A (*k_a_* = 95.9 and 64.2 m^−1^ s^−1^, respectively). The structural integrity of this K72A derivate was previously confirmed by circular dichroism spectroscopy (18). This indicates that Lys-72 appears to be required for efficient G-actin binding and suggests the residue may form part of the G-actin-binding site. The only determined crystal structure of an ADF/cofilin in complex with actin (that of the non-severing mouse N-terminal Twinfilin-ADF homology domain), however, found the equivalent residue (serine 252) on the opposing face of the Twinfilin molecule directly facing away from the actin monomer (12) (Fig. 1*F*). This suggests that PfADF1 may interact with G-actin in a different manner to that observed in the non-severing Twinfilin.

##### Chemical Cross-linking with Mass Spectrometry Reveals a Novel G-actin-PfADF1 Binding Interface

**FIGURE 2.**
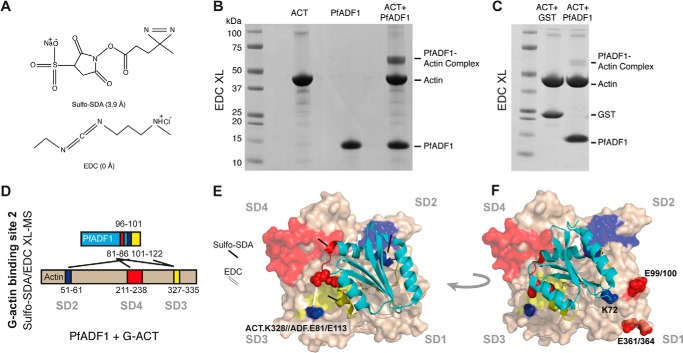
**The structural basis for PfADF1 and G-actin interactions determined by XL-MS.** *A*, chemical structures of cross-linkers. Sulfo-SDA cross-links lysine to a non-selective amino acid, EDC cross-links lysine to an acidic amino acid (Asp or Glu). *B–D*, SDS-PAGE gels showing the migration of (*B*) covalently linked stable complex of monomeric (G)-actin-PfADF1 using EDC and (*C*) free actin mixed with either GST control or PfADF1 (note between 10 and 25% of actin and PfADF1 are linked by EDC, which is in agreement with their low affinity to form complex). *D*, interaction between PfADF1 and G-actin analyzed by sulfo-SDA and EDC XL-MS. Three sets of detected sulfo-SDA- and two sets of EDC cross-linked peptides are colored in *red*, *yellow*, and *blue* as highlighted in their primary structures. *E*, reconstructed XL-MS actin-PfADF1 structural model combining data from cross-linkers sulfo-SDA and EDC. Cross-linked sites by EDC and sulfo-SDA are indicated by *white* and *black lines*, respectively. Domains involved in cross-linked peptides are colored (as *D*). Lysine and acid residues are shown in *blue* and *red spheres*, respectively. *F*, reconstructed G-actin·PfADF1 complex showing basic residue Lys-72 from PfADF1 and acidic residues Glu-99, -100, -361, and -364 from actin. Basic and acidic residues are indicated as *blue* and *red spheres*, respectively. Coloring of the G-actin and PfADF1 models and the cross-linked peptides are as described above.

Attempts to co-crystallize PfADF1 bound to monomeric actin were unsuccessful. To gain a detailed understanding of how PfADF1 interacts with and severs actin filaments, we therefore determined the structural basis of the G-actin-PfADF1 interaction at residue resolution by chemical XL-MS (26–31). This method compliments crystallographic approaches, in that it is able to capture transient interactions and was thus well suited to PfADF1, given its relatively low affinity for actin (Fig. 1*E*). Recombinant PfADF1 preincubated with monomeric rabbit skeletal muscle actin in the presence of the zero length cross-linking agent 1-ethyl-3-(dimethylaminopropyl)carbodiimide (EDC) yielded a G-actin·PfADF1 complex of ∼56 kDa (Fig. 2, *A* and *B*). Incubation of actin with glutathione *S*-transferase (GST) in the presence of EDC under the same conditions did not yield a protein complex (Fig. 2*C*). EDC covalently links lysine and acidic residues (Glu or Asp) via a covalent isopeptide bond between the ϵ-amine of lysine and carboxylate group of the acidic residue, allowing fine mapping of interactions mediated by salt bridges. Cross-linked bands were excised, subjected to in-gel tryptic digestion, and analyzed by UPLC and high resolution MS/MS on a Q-Exactive mass spectrometer to detect linked peptides between PfADF1 and actin with the peptide identity searched using a recently developed algorithm to identify cross-linked peptides based on false discovery rate estimation (22). Given the nature of the EDC cross-links, manual validation of the high-resolution spectra permitted mapping of salt bridge interactions between the actin and PfADF1 crystal structures (PDB codes 1J6Z and 3Q2B) with near residue resolution (18, 32). Specifically, SD3 (Lys-328; peptide-(327–335)) of actin was found cross-linked with the coil region preceding α4 (Glu-113; peptide-(101–122)) of PfADF1 (Fig. 2*D*). In addition, SD3 (Lys-328; peptide-(327–335)) of actin was found cross-linked with the coil region preceding α3 (E81; peptide-(81–86)) of PfADF1 (Fig. 2*D*). The peptide coverage encompassed most of the PfADF1 C-terminal regions spanning the α3 helix to coil-β6-α4 domains (Fig. 2*D*). Representative spectra are shown in supplemental data (see supplemental Fig. S1, *A* and *B*). Of note, we did not detect any spurious inter-protein cross-links supporting the specificity of this method. Additionally, no peptide or cross-linked identifications were reported when searched with the reversed protein XComb generated database. To further corroborate EDC, we also used sulfo-NHS-diazirine (sulfo-SDA), a short cross-linking agent (3.9 Å), which links lysine residues to non-selective groups (Fig. 2*A*). Sulfo-SDA cross-linked peptides showed strong spatial overlap to those found with EDC as well as detecting additional cross-linked species. Sulfo-SDA cross-linking revealed PfADF1 residues 81–86, 96–101, and 101–122 bound to subdomain 4 (SD4) (peptide-(211–238)), SD2 (peptide-(51–61)), and SD3 (peptide-(327–335)) of the actin monomer, respectively (Fig. 2*D*, supplemental Fig. S1, *C-E*, Table 1, and supplemental Table S1).

**TABLE 1:** **Summary of G-actin-PfADF1 peptides detected**

Actin peptide Uniprot ID: P68135	PfADF1 peptide Uniprot ID: Q81467	Site of cross-link	Cross-link agent	Δ*m*/*z* error
				*ppm*
Peptide-(327–335) (IKIIAPPER)	Peptide-(81–86) (ESSNSR)	PfADF1.E81	EDC	0.707
		Actin.K328		
Peptide-(327–335) (IKIIAPPER)	Peptide-(101–122) IEGVNVLTSVIESAQDVADL	PfADF1.D117/D120	EDC	0.759
		Actin.K328		
Peptide-(211–238) (DIKEKLCYVALDFENEMATAASSSSLEK)	Peptide-(81–86) (ESSNSR)	Actin.K215	SDA	−2.963
Peptide-(51–61) (DSYVGDEAQSK)	Peptide-(96–101) (QAILKK)	PfADF1.K100	SDA	−1.630
Peptide-(327–335) (IKIIAPPER)	Peptide-(101–122) (IEGVNVLTSVIESAQDVADL)	Actin.K328	SDA	−0.347

Combining EDC and sulfo-SDA cross-links enabled us to model the G-actin-PfADF1 interaction using their two crystal structures (PDB codes 1J6Z and 3Q2B) (18, 32) (Fig. 2*E*). The reconstructed structural model shows PfADF1 in contact with all four subdomains of the actin monomer via a previously uncharacterized interaction interface. Significantly, this interaction is markedly different from the previously determined crystal structure of the mouse Twinfilin ADFH·actin complex (12). In this complex, the N terminus (residues 176–181), long α3 helix (residues 266–274), and distal coil region (residues 294–302) would be expected to fit into the groove between SD1 and SD3 of G-actin (Fig. 1*F*). Although a cross-linked PfADF1 peptide containing the Lys-72 severing residue was not detected by either cross-linking chemistry, the modeled actin·PfADF1 complex structure unambiguously demonstrates that Lys-72 would contact SD1 of G-actin, potentially via interactions with several acidic residues (Glu-99, -100, -361, or -364) within SD1 (Fig. 2*F*). This would explain the low affinity binding of the mutant seen in sedimentation and surface plasma resonance binding assays.

##### PfADF1 Binds to F-actin via the Novel Decoration-independent Interface

**FIGURE 3.**
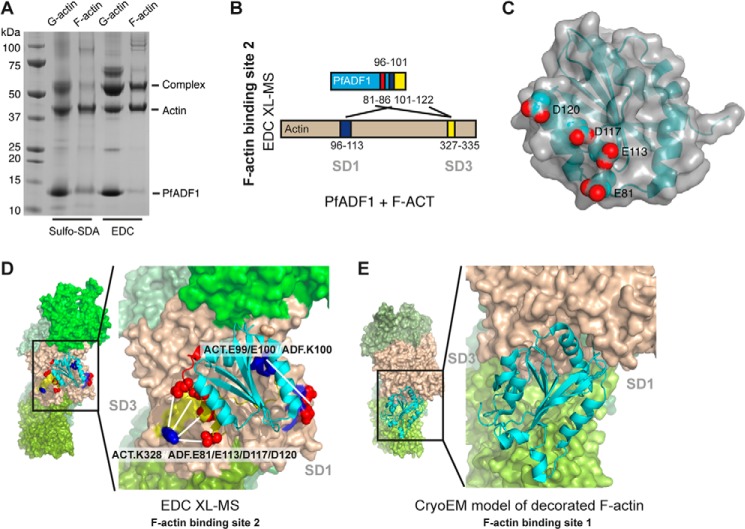
**The structural basis for PfADF1 and F-actin interactions determined by XL-MS.** *A*, an SDS-PAGE gel showing migration of the covalently linked stable complex of either monomeric (G)- or filamentous (F)-actin with PfADF1 using sulfo-SDA and EDC cross-linkers. *B*, interaction between PfADF1 and F-actin determined by EDC XL-MS. Three sets of detected EDC-cross-linked peptides corresponding to actin-binding site 2 are colored in *red*, *yellow*, and *blue* as highlighted in their primary structures. *C*, surface representation of PfADF1 showing an acidic residue cluster (*red spheres*) predicted to interact with F-actin. *D*, structural model of PfADF1 binding to F-actin at the novel interface as derived from EDC XL-MS data (three sets of cross-linked peptides are colored as *B*). Lysine and acid residues are shown in *blue* and *red spheres*, respectively. EDC-cross-linked sites are indicated by *white lines. E*, published cryo-EM structure of the HsCOF2-decorated actin filament showing the decorative mode of interaction (actin-binding site 1).

Having explored interactions with monomeric actin, we next sought to determine the molecular basis for F-actin-PfADF1 interactions by XL-MS following incubation of PfADF1 with pre-formed actin filaments. Cross-linked species were readily detected using either sulfo-SDA or EDC cross-linkers (Fig. 3*A*). Using the higher resolving power of EDC, cross-linked peptides corresponding to three sites of F-actin-PfADF1 interaction were detected (PfADF1 peptides-(81–86), -(96–101), and -(102–122), and actin peptides-(327–335), -(96–113), and -(327–335), respectively) (supplemental Fig. S2, *A-C*, Table 2, and supplemental Table S1). At the residue level, SD3 (Lys-328) of actin was found cross-linked with both α4 (all Glu-113, Asp-117, and Asp-120 sites appeared to be present in the cross-linked spectra) and Glu-81 of PfADF1 (Fig. 3*B*). Glu-113, Asp-117, Asp-120, and Glu-81 form a highly acidic patch on the face of PfADF1 (Fig. 3*C*), which indicates that alternative acidic residues of PfADF1 may be interchangeably used to form salt bridges with SD3 of F-actin. In addition, SD1 (Glu-99 or Glu-100) of actin was found cross-linked with α3 (Lys-100) of PfADF1. Reconstructing the F-actin-PfADF1 structural model based on the F-actin-PfADF1 XL-MS data shows that PfADF1 interacts with F-actin using an interface that overlaps with that involving G-actin (Figs. 2, *D-F*, and 3*D*). The overlapping regions of G- and F-actin include PfADF1 peptide-(102–122) linked to the actin SD3 peptide-(327–335). Collectively, detection of cross-linked peptides and the XL-MS-derived structural models reveals that PfADF1 interacts with actin (either in a G or F state) via a new interaction interface, which we call actin-binding site 2, distinct from the expected canonical decorative interaction (actin-binding site 1) (Fig. 3*E*).

**TABLE 2:** **Summary of F-actin-PfADF1 peptides detected**

Actin peptide Uniprot ID: P68135	PfADF1 peptide Uniprot ID: Q81467	Site of cross-link	Cross-link agent	Δ*m*/*z* error
				*ppm*
Peptide-(327–335) (IKIIAPPER)	Peptide-(81–86) (ESSNSR)	PfADF1.E81	EDC	1.207
		Actin.K328		
Peptide-(96–113) (VAPEEHPTLLTEAPLNPK)	Peptide-(96–101) (QAILKK)	PfADF1.K100	EDC	1.093
		Actin.E99/100		
Peptide-(327–335) (IKIIAPPER)	Peptide-(101–122) (IEGVNVLTSVIESAQDVADL)	PfADF1.D117/120	EDC	−0.687
		Actin.K328		

Direct testing of XL-MS using PfADF1 with its native actin was precluded by the low levels of *in vitro* expression for PfActin. However, sequence alignment of *Plasmodium* and rabbit actins clearly demonstrate that the residues involved in the novel actin-binding site are entirely conserved, namely actin residues Glu-99, Glu-100, and Lys-328. Thus, whereas PfActin is known to have divergent properties (most relevantly its inherent instability (33)), we predict the novel interaction of PfADF1 is entirely conserved in the native parasite context.

##### XL-MS Validates the Canonical F-actin-HsCOF1 Decorative Interaction

**FIGURE 4.**
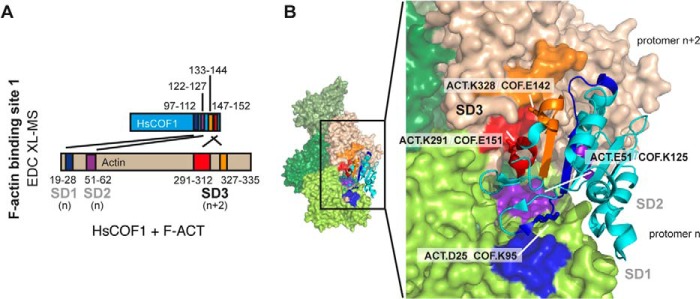
**The structural basis for HsCOF1-F-actin interaction determined by EDC XL-MS.** *A*, interaction between HsCOF1 and F-actin at the decoration site determined by EDC XL-MS. Four sets of detected EDC-cross-linked peptides corresponding to actin-binding site 1 are colored in *blue*, *purple*, *red*, and *orange* as highlighted in their primary structures. *B*, four sets of EDC cross-linked F-actin-HsCOF1 peptides were mapped onto the cryo-EM structure of the HsCOF2 decorated filament. Colors are as described in *A*. Cross-linked sites are indicated by *white lines*.

We then attempted to use XL-MS to characterize the interaction between F-actin and a conventional ADF/cofilin, HsCOF1. EDC XL-MS of HsCOF1 bound to F-actin revealed four cross-linked peptides corresponding to the decorative interaction interface as described in detail previously (9) and entirely consistence with the sites of interactions observed in the cryo-EM structure of the F-actin·HsCOF2 complex (9). Specifically, SD3 of actin protomer *n*+2 (Lys-328 from peptide-(327–335) and Lys-291 from peptide-(291–312)) were found interacting with Glu-142 (peptide-(133–144)) and Glu-151 (peptide-(147–152)) of HsCOF1, respectively (Fig. 4, *A* and *B*, supplemental Fig. S3, *A* and *B*, Table 3, and supplemental Table S1). For actin protomer *n*, HsCOF1 was found to interact with Asp-25 of SD1 (peptide-(19–28)) via Lys-95 (peptide-(93–112)), whereas Glu-51 of SD2 (peptide-(51–62)) was found to interact with Lys-125 (peptide-(122–127)) (Fig. 4, *A* and *B*, supplemental Fig. S3, *A-E*, Table 3, and supplemental Table S1).

**TABLE 3:** **Summary of F-actin-HsCOF1 peptides detected**

Actin peptide Uniprot ID: P68135	HsCOF1 peptide Uniprot ID: P23528	Site of cross-link	Cross-link agent	Δ*m*/*z* error
				*ppm*
Peptide-(327–335) (IKIIAPPER)	Peptide-(133–144) (HELQANCYEEVK)	HsCOF1.E142	EDC	−2.331
		Actin.K328		
Peptide-(291–312) (KDLYANNVMSGGTTMYPGIADR)	Peptide-(147–152) (CTLAEK)	HsCOF1.E151	EDC	1.593
		Actin.K291		
Peptide-(19–28) (AGFAGDDAPR)	Peptide-(93–112) (ESKKEDLVFIFWAPESAPLK)	HsCOF1.K95	EDC	−3.190
		Actin.D25		
Peptide-(51–62) (DSYVGDEAQSKR)	Peptide-(122–127) (DAIKKK)	HsCOF1.K125	EDC	−1.329
		Actin.D51		
Peptide-(51–61) (DSYVGDEAQSK)	Peptide-(122–127) (DAIKKK)	HsCOF1.K125	EDC	−1.040
		Actin.D51		

Taken together, these cross-linked peptides provided an accurate map of HsCOF1 decoration of actin entirely consistent with cryo-EM observations from HsCOF2 (9). Moreover, our cross-link data using HsCOF1-decorated F-actin correlate well with a previous study in which cofilin was detected cross-linked with the cleft between SD1 and SD3 of G-actin (34). Furthermore, intra-subunit cross-linked peptides were detected for both actin and HsCOF1 (Table 4 and supplemental Table S1), which correlate with the determined crystal structure of G-actin and the NMR structure of HsCOF1.

**TABLE 4:**
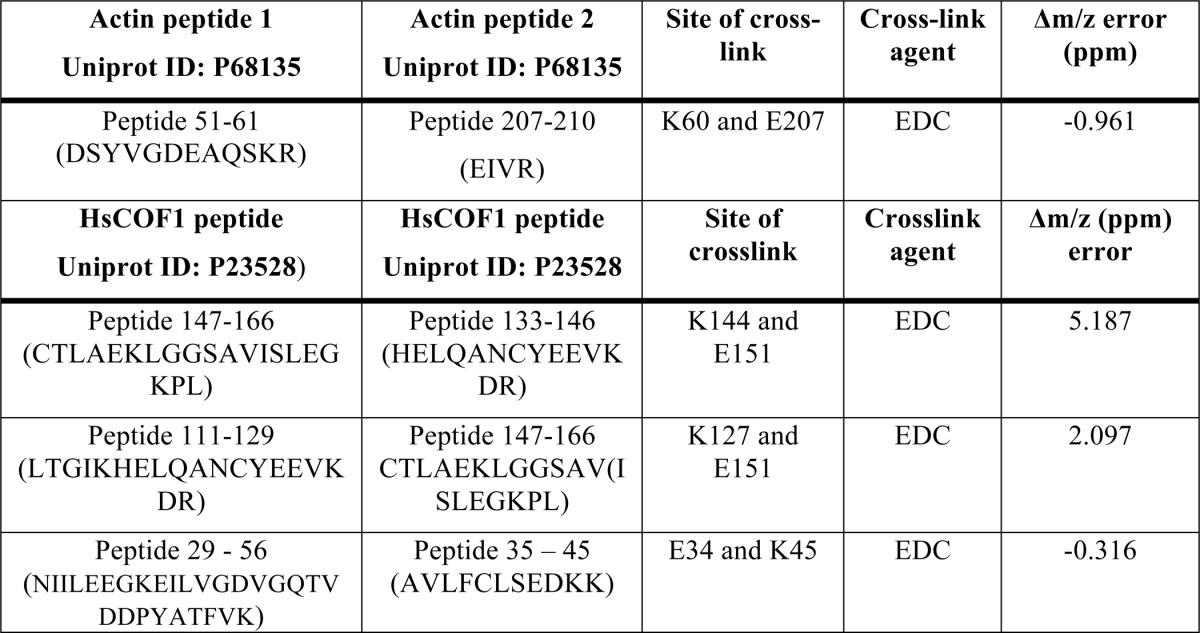
**Summary of intra cross-linked actin and ADF/cofilin peptides detected**

Despite multiple attempts, we did not detect any HsCOF1-F-actin peptide corresponding to the novel actin-binding site 2 found for the F-actin-PfADF1 interaction. This may not be surprising given that HsCOF1 decoration of F-actin would be expected to saturate interactions with actin at high concentrations (dominating spectra). Furthermore, once bound, decoration would sterically block the novel binding site due to their overlapping nature.

##### Selective Inhibition of F-actin Decoration Enhances HsCOF1-mediated Severing

**FIGURE 5.**
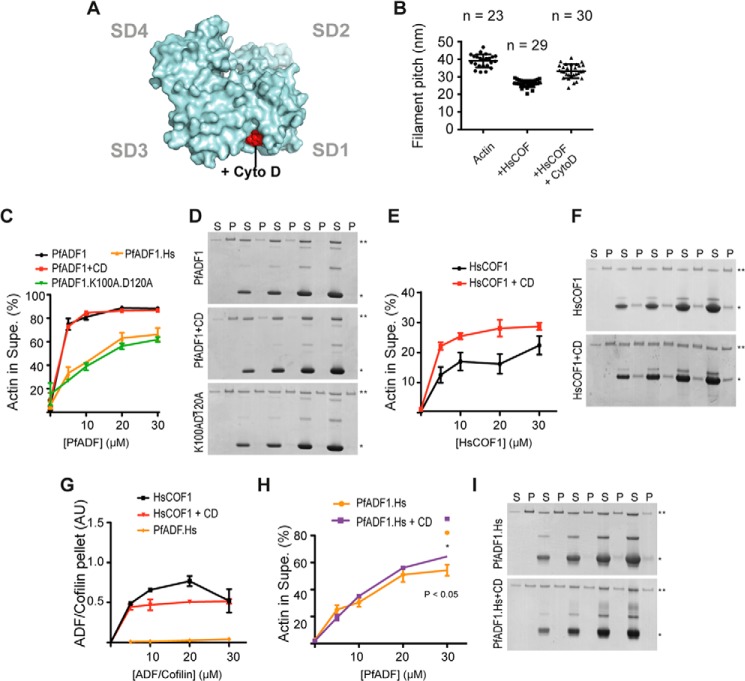
**Blocking actin-binding site 1 reduces filament decoration and decreases filament sedimentation in HsCOF1 but not PfADF1.** *A*, model of the published crystal structure of actin in complex with CD (PDB 3EKU; Ref. 26). CD is shown as *red spheres. B*, measurement of filaments pitch by TEM (mean ± S.E.). *C*, depolymerization of F-actin by PfADF1 derivatives measured by sedimentation analysis with short actin species (supernatant) shown proportionally to intact filaments (pellet) analyzed by densitometry (*n* = 3, mean ± S.E.). *D*, representative gels from *C* where symbols represent: *, actin; **, ADF/cofilin; *P*, pellet; and *S*, supernatant. *E*, depolymerization of F-actin by HsCOF1 in the absence or presence of CD measured by sedimentation analysis. *F*, representative gels from *E. G*, co-sedimentation of ADF/cofilins with F-actin. The amount of ADF/cofilin in the pellet was analyzed by densitometry, normalized against CD treatment (*e.g.* see *lanes 1* and *2* of *F*) with F-actin alone (*n* = 3, mean ± S.E.). *H*, depolymerization of F-actin by the PfADF1.Hs chimera measured by sedimentation analysis in the absence or presence of CD (*n* = 3, mean ± S.E.). *Asterisk* denotes *p* < 0.05 using Student's *t* test. Representative gels are from *H*.

In the absence of direct proteomic evidence, we sought an alternative approach to test whether it was possible to achieve severing independent of decoration via blockage of the SD1-SD3 decoration site in a canonical ADF/cofilin. Recent evidence has demonstrated that CD, a fungal-derived small organic actin inhibitor, is able to competitively inhibit the interaction between human cofilin and actin in both F and G states (35). The crystal structure for actin bound to CD has been solved (36) and involves the same hydrophobic cleft between SD1 and SD3 as that bound in the Twinfilin-ADFH-actin structure (12) (Fig. 5*A*). We therefore reasoned that incubation of F-actin with CD should block the decoration site and explain the ability of the inhibitor to compete with cofilin (35). TEM showed that HsCOF1 reduced the pitch of the actin filament helix from 40 to 26.3 nm (*n* = 29), whereas the pitch was intermediate (33.2 nm, *n* = 30) with HsCOF1 in the presence of 2-fold molar excess of CD (Fig. 5*B*). This supports the notion that the HsCOF1 decoration and CD binding sites are conserved. Absence of decoration with PfADF1 precluded testing of a CD effect in this context.

Actin sedimentation analyses were also undertaken with both ADF/cofilins and preformed actin filaments in the presence of CD. Increasing concentrations of PfADF1 resulted in an increased amount of actin in the supernatant (monomers, actin filament nuclei, or short filaments, which we refer to collectively as short filaments) in a concentration-dependent manner as expected (85% of actin found in the supernatant at 30 μm PfADF1; Fig. 5, *C* and *D*). The amount of short filaments generated by HsCOF1 under the same conditions was considerably lower and appeared to reach a plateau at ∼30 μm (20% actin found in the supernatant; Fig. 5, *E* and *F*) consistent with the filament reaching a saturated decoration state, where severing is protected. Of note, a proportion of HsCOF1 but not PfADF1 co-pelleted with F-actin (Fig. 5, *D*, *F*, and *G*), consistent with their respective abilities to decorate and stabilize filaments (Fig. 1*A*). The presence of 1 μm CD had no effect on the ability of PfADF1 to generate short filaments (Fig. 5, *C* and *D*). However, incubation with HsCOF1 in the presence of the same concentration of CD demonstrated a marked increase in the amount of short filaments generated (Fig. 5, *E* and *F*). We have previously generated a chimera between PfADF1 and HsCOF1, which contains an extended F-loop and full α4 helix (PfADF1.Hs) that severs filaments effectively (18). Sedimentation assays with chimeric PfADF1.Hs demonstrated a lower amount of short filaments generated than wild type (Fig. 5, *C*, *H*, and *I*). This is readily explained by a small, but significant increase in the ability of the chimera to co-sediment with filaments at high micromolar concentrations, which is absent in wild type ADF1 (Fig. 5, *D* and *I*) (18). Given the gain in ability to bind filaments, we reasoned that PfADF1.Hs would also be sensitive to CD. As predicted, treatment of PfADF1.Hs with CD led to a small but significant increase in the amount of short filaments generated compared with untreated assays (Fig. 5, *H* and *I*). It is also noteworthy that under the same conditions, co-sedimentation with filamentous actin in the pellet was ablated (Fig. 5*I*).

**FIGURE 6.**
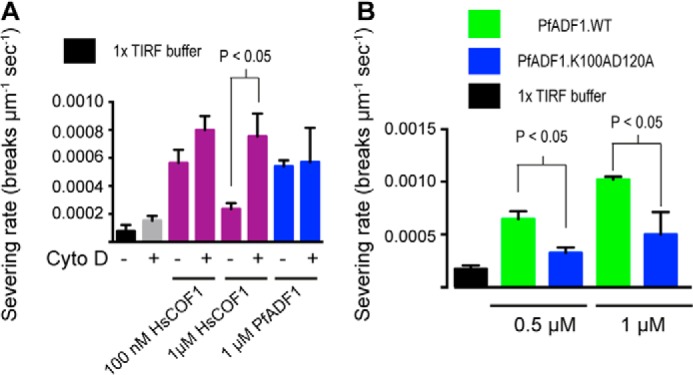
**Blocking actin-binding site 1 leads to an increase in filament severing rate in HsCOF1 but not PfADF1.** *A*, TIRF microscopy measurements of F-actin severing by HsCOF1 and PfADF1 in the presence/absence of CD (see also supplemental Movies S1–S4) (*n* = 3, mean ± S.E.). Comparison denotes *p* < 0.05 using Student's *t* test. *B*, TIRF microscopy measurement of F-actin severing by wild type PfADF1 and PfADF1.K100AD120A mutant (*n* = 3, mean ± S.E.). Comparison denotes *p* < 0.05 using Student's *t* test.

To complement the sedimentation assay, HsCOF1 severing of F-actin in the absence or presence of CD was tested by TIRF microscopy, providing visualization of single actin filament dynamics. Although CD treatment had no effect on F-actin severing rates under the conditions tested, actin filament severing by HsCOF1 was 2.5-fold higher in the presence of CD compared with the untreated control (Fig. 6*A*, supplemental Movies S3 and S4). This indicates that inhibiting decorative actin-binding site 1 skews HsCOF1 toward enhanced severing. Treatment of F-actin with CD had no effect on the severing rate of PfADF1 as expected (Fig. 5*C*). Combined these data demonstrate that CD directly competes with the filament decoration site and promoted HsCOF1-mediated severing, suggesting it may also interact with actin-binding site 2 to mediate severing.

##### Actin-binding Site 2 Is Required for PfADF1-mediated Severing

To directly test implicated residues of PfADF1 in severing via the novel actin-binding site, we made alanine substitutions at two sites found using XL-MS that are not predicted to be involved in a decorative-like interaction (Fig. 3*B*). Mutation of Lys-100 and Asp-120 of PfADF1, which map to the SD1 and -3 interactions with F-actin showed a marked reduction in the ability of PfADF1 to generate short filaments by sedimentation (Fig. 5*C*). Complementary analysis showed these residues are required for severing as measured by TIRF microscopy (Fig. 6*B*). Thus, this demonstrates that critical residues that map to the novel actin-binding site 2 are required for PfADF1-mediated severing of F-actin. The proximity of residues in HsCOF1 decorative interactions and those predicted to be involved in the novel binding site (Fig. 4*C*) precluded similar mutation studies in HsCOF1. Combined these data demonstrate that PfADF1 likely severs actin via a novel interaction interface and also suggests that this mechanism may be widely conserved across ADF/cofilin family members.

## DISCUSSION

In this study, XL-MS combined with protein complex structural reconstruction has enabled us to identify and characterize a second actin-binding site for the malaria parasite ADF/cofilin, PfADF1. The functionality of this site has been validated by the actin filament sedimentation assay as well as via single molecule imaging using TIRF microscopy revealing that this novel actin-binding site is likely required for F-actin severing by PfADF1. Interestingly, biochemical analyses showed that selective inhibition of decoration led to an enhanced level of F-severing by HsCOF1. Two possibilities can explain this observation. First, ADF/cofilins are more efficient in F-actin severing at low-density interactions with the actin polymer (6). Because chemical inhibition of decoration would reduce the effective concentration of HsCOF1 available to interact with F-actin, this might have concomitantly led to enhanced severing. A second interpretation, which we favor here, is that severing of F-actin by ADF/cofilins may be entirely independent of canonical decoration and alternatively achieved via ADF/cofilins binding to a novel actin-binding site as demonstrated for PfADF1. At present this is difficult to prove experimentally for HsCOF1, because the primary mode of F-actin-HsCOF1 decorative interaction overlaps with and, thus, precludes cross-linking detection of the novel site. However, if validated, we believe that diverse ADF/cofilins may also interact with the actin polymer via two actin-binding sites, a canonical site (actin-binding site 1) used for F-actin decoration and a second actin-binding site (site 2) identified in this study, which defines the molecular basis for severing. Critically, based on the XL-MS identified inter-protein contact residues and reconstructed structural models (Figs. 2–4), occupancy of the two binding sites would be expected to be mutually exclusive for a canonical ADF/cofilin. This may help to explain the molecular basis for ADF/cofilin protein multifunctionality and predict the modes of action of ADF/cofilins from divergent eukaryotic species.

**FIGURE 7.**
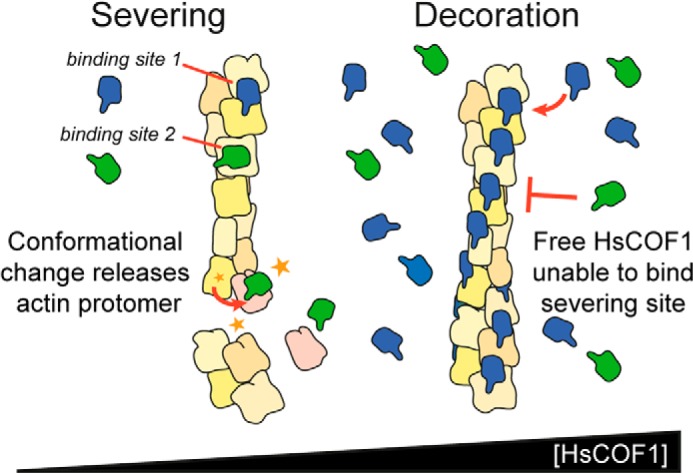
**A revised model for regulation of actin filament dynamics by ADF/cofilins via two interaction interfaces.** A bimodal model for canonical ADF/cofilin interactions with the actin filament based on human COF1. At low (nanomolar) HsCOF1 concentrations filaments are exposed, with both decoration (actin-binding site 1) and severing sites (actin-binding site 2) in the filament accessible. Irrespective of the affinity of the ADF/cofilin for the two sites, the number of potential sites along the filament means that severing (green surface of HsCOF1 up) is highly favored. At high (micromolar) HsCOF1 concentrations, filaments are decorated (*blue surface* of HsCOF1 up). This blocks the severing site and the actin filament is therefore stable and severing is strongly inhibited.

In apicomplexan parasites, in the absence of the long filament binding loop/α4 motif and canonical decorative interaction, site 2 is the only site that produces a functional, productive effect on filament dynamics. Thus, the polymer twisting/stabilizing potential of apicomplexan ADFs is lost, resulting in functional reduction toward a single mode of interaction, *i.e.* severing. In contrast, canonical eukaryote ADF/cofilins (such as HsCOF1) would be predicted to display a bimodal function with respect to filament dynamics, being able to both decorate and sever, with the latter only possible in undecorated and exposed regions of filamentous actin. This provides a logical explanation to the ADF/cofilin concentration-dependent functionality (6) and is also consistent with observations that severing occurs preferentially at boundaries between decorated and exposed regions of the filament (11). This proposed model (shown in Fig. 7) therefore interprets the multiple effects of ADF/cofilin proteins on F-actin dynamics as due to the presence of two actin-binding sites, whereby F-actin stabilization and severing are dictated by the site of occupancy, the balance of affinity for the two interaction sites, the local cellular concentrations of ADF/cofilin (6) and (if relevant) its phosphorylation state (2).

An important feature of our model is that it can explain the observation that at very low concentrations (*i.e.* low density binding to F-actin), a small number of molecules are sufficient to destabilize the filament lattice and promote severing (37, 38). In apicomplexan parasites, the divergent properties of parasite ADF proteins on filament dynamics ensure that F-actin-severing sites are always exposed due to the elimination of canonical decoration and, as such, result in rapid and constant turnover of actin filaments (39).

Independent support for the second binding site can be seen in previous mutational studies undertaken with the yeast ADF/cofilin ScCOF (40). Specifically, residues Asp-10, Glu-11, Asp-34, Lys-36, and Glu-38 of ScCOF were shown to be involved in F-actin severing, but not F-actin binding (40). Asp-34, Lys-36, and Glu-38 of ScCOF are facing the same side as the face of PfADF1 involved in severing. This lends weight to the suggestion that canonical F-actin binding via site 1 and F-actin severing via site 2 may be indeed be two separate events and may be widely conserved across ADF/cofilins.

A remaining challenge for the model is the precise structural basis for the novel interaction. Based on XL-MS data two residues of PfADF1 (Glu-81 and Lys-100 of 27 Å apart) reach two actin residues (Glu-100 and Lys-328) that are separated by as much as 49 Å as measured by their respective crystal structures, which has a prominent convex actin surface to it. To accommodate the novel interaction, both PfADF1 and actin would need to undergo substantial conformational changes for these residues to form close contact. Structural polymorphism within the actin polymer are, at least, well documented (41). It is alternatively possible that PfADF1 may bind actin via one site before forming contact with the other site (effectively rocking between the two). Ultimately, definitive demonstration of the structural basis of severing will require isolation of crystallographic or cryo-EM structural intermediates of severing and/or further dual color TIRF imaging of mutant non-decorating, but severing competent ADF/cofilins.

One intriguing question is why the apicomplexan ADF/cofilin proteins have lost their ability to decorate actin filaments. One hypothesis is that malaria and related apicomplexan parasite ADF proteins have evolved to help maintain very short filaments (39, 42, 43) with rapid turnover (39), thus losing their ability to stabilize F-actin. In support of this notion, introduction of mutations that stabilize the filament are severely detrimental to apicomplexan parasite viability (33). In addition these parasites have almost entirely lost all known actin filament-binding proteins (44, 45). Thus actin regulation in Apicomplexa appears to be geared toward filament severing, creating a selective pressure to reduce the filament stabilizing activity of the ADF/cofilin proteins present. This places PfADF1 and its apicomplexan orthologues in a central role mediating filament turnover (15).

The identification of the second ADF/cofilin F-actin-binding site by XL-MS highlights a critical benefit this technique has for structural and biochemical characterizations of transient protein-protein interactions such as those that underpin actin regulation. This is in contrast to bulk actin assays like that using Pyrene-actin or static structural techniques like cryo-EM, which has defined much of our structural understanding of actin filament biology to date (*e.g.* Refs. 9 and 41)). Although cryo-EM in particular is excellent in providing near atomic details of the decorating interaction in a complex like the F-actin-cofilin polymer, low affinity or transient interactions such as those involving actin-binding site 2 are unlikely to be detected. This demonstrates the great utility of XL-MS (26–28), combined with molecular modeling, as an analytical approach to structurally characterize low affinity protein complexes that are otherwise difficult to prepare for analysis by standard techniques such as x-ray crystallography or cryo-EM.

## Footnotes

5 The abbreviations used are:

ADF: actin deploymerizing factor
PfADF1: *Plasmodium falciparum* ADF1
HsCOF: human cofilins
XL-MS: chemical cross-linking coupled mass spectrometry
TIRF: total internal reflection fluorescence
CD: cytochalasin D
TEM: transmission electron microscopy
EDC: 1-ethyl-3-(dimethylaminopropyl)carbodiimide
sulfo-SDA: sulfo-NHS-diazirine
PDB: Protein Data Bank
SD: subdomain(s).
